# Estimating the Number of Low-Income Americans Exposed to Household Air Pollution from Burning Solid Fuels

**DOI:** 10.1289/ehp.1306709

**Published:** 2014-05-09

**Authors:** Derek K. Rogalsky, Pauline Mendola, Tricia A. Metts, William J. Martin

**Affiliations:** 1 Georgetown University School of Medicine, Georgetown University, Washington, DC, USA; 2 *Eunice Kennedy Shriver* National Institute of Child Health and Human Development, National Institutes of Health, Department of Health and Human Services, Bethesda, Maryland, USA; 3 Department of Environmental Health, East Tennessee State University, Johnson City, Tennessee, USA

## Abstract

Background: Exposure to household air pollution (HAP) from inefficient biomass and coal stoves kills nearly 4 million people every year worldwide. HAP is an environmental risk associated with poverty that affects an estimated 3 billion people mostly in low- and middle-income countries.

Objectives: Our goal was to estimate the number of low-income Americans exposed to potentially health-damaging concentrations of HAP.

Methods: We mapped county-level data for the percentage of households using wood, coal, and/or coke as their primary heating fuel along with percent of the population below the federal poverty level. Using U.S. Census data and the likelihood of fugitive emissions as reported in the literature, we estimated the number of low-income Americans potentially exposed to HAP.

Results: Solid fuel is the primary heating source for > 2.5 million U.S. households, or 6.5 million people. The mapping exercise showed several rural areas, primarily in the northern and western regions, that have high levels of solid-fuel use and poverty. We then identified 117 counties with high co-incident poverty and solid-fuel use as high-priority counties for research into potential health risks from HAP. We estimate that between 500,000 and 600,000 low-income people in the United States are likely exposed to HAP from burning solid fuels within their homes.

Conclusion: HAP occurs within the United States and should be further investigated for adverse health risks, especially among those living in areas with rural poverty.

Citation: Rogalsky DK, Mendola P, Metts TA, Martin WJ II. 2014. Estimating the number of low-income Americans exposed to household air pollution from burning solid fuels. Environ Health Perspect 122:806–810; http://dx.doi.org/10.1289/ehp.1306709

## Introduction

Worldwide, 3 billion people rely on burning biomass and other solid fuels for cooking and heating within their homes (World Bank 2011). Each year, exposure to household air pollution (HAP) from inefficient stoves kills 3.5 million people directly, as well as another 0.5 million people from HAP’s contribution to outdoor air pollution (Lim et al. 2012). In general, these people live in extreme poverty within low- and middle-income countries (LMIC), and procuring fuel for heating and cooking consumes much of their time and resources (World Bank 2011). HAP is an independent risk factor for low birth weight, childhood pneumonia, chronic obstructive pulmonary disease (COPD), cataracts, cardiovascular disease, and lung cancer [Boy et al. 2002; Kurmi et al. 2010; Lee et al. 2012; McCracken et al. 2011; Pope et al. 2010; Smith et al. 2000; World Health Organization (WHO) 2009; Zhang and Smith 2007; Zhong et al. 2007]. Other outcomes have been proposed, but the evidence is less definitive for asthma, cancers other than lung, pneumonia in adults, and infectious diseases such as tuberculosis or HIV (Fullerton et al. 2008; Smith et al. 2004). Awareness of these health risks has sparked a global effort to have 100 million households adopt clean cooking technologies by 2020 (Martin et al. 2011; United Nations Foundation 2012).

The problem of HAP is less often studied in developed countries. However, a growing body of literature suggests that despite the relative affluence of developed countries, the rural poor in the United States, and perhaps elsewhere, are at risk due to HAP exposure in much the same way as occurs globally (Barry et al. 2010; Bulkow et al. 2012; Bunnell et al. 2010; Johnston et al. 2013; Noonan et al. 2012b). HAP most often results from the daily inefficient combustion of fuels indoors without sufficient ventilation to remove emissions (Lim et al. 2012; World Bank 2011). The same principle holds for HAP in developed countries where “fugitive emissions”—products of incomplete combustion that escape the stove or flue—pollute the indoor environment. However, important differences exist between HAP globally and within the United States (Naeher et al. 2007). Namely, very few stoves within the United States function without a flue, and most stoves are used for heating seasonally as opposed to year-round cooking. Also, the duration in which children are exposed to HAP is likely lower in the United States, where children are most often in school for a significant portion of the day during the heating season. Although these differences change the type and duration of exposure, significant health risks may remain. Globally, HAP is tightly linked with poverty because families have limited financial resources to spend on more efficient stoves or fuels (World Bank 2011). In this study, we sought to determine areas of the United States where poverty and household solid-fuel use coexist, and to provide an estimate of the number of low-income Americans at risk from fugitive emissions escaping from the indoor stoves. To quantify the potential scope of the problem, we used publicly available data sets to estimate the number of households in the United States with coincident primary solid-fuel use and low income. We then used the literature to estimate the likelihood of fugitive emissions in such households and calculated the number of people living in poverty in the United States who could be at risk for poor health from HAP.

## Methods

To determine the number of households that use solid fuels as their primary heating source and live below the federal poverty level (FPL) in the United States, we queried the U.S. Census Public Use Microdata Sample (PUMS) for the American Community Survey (ACS) 5-year estimate 2006–2010 (U.S. Census Bureau 2012). The PUMS of the ACS provides national and statewide estimates of households that use solid fuels as a primary heat source. The FPL is defined by using set income levels adjusted for inflation and family size; for example, the FPL in 2011 for a family of four with two children was $22,811 (U.S. Census Bureau 2012). Data on poverty and the rural–urban continuum score (year 2004) came from the Economic Research Service of the U.S. Department of Agriculture (USDA) (2012). We combined these data using the Federal Information Processing Standards (National Institute of Standards and Technology 2012) codes and imported the data into the mapping software Health Landscape (Health Landscape 2012). With the mapping software, the county-level data were then displayed using a geographic boundary file for 3,144 counties and county equivalents in the United States in 2010. The county-level rates for solid-fuel use and poverty were assigned to nine equal quantiles and color coded using Health Landscapes, which allows information that has been geocoded by coordinates or to state, county, or local boundaries to be displayed on preset maps.

To identify priority counties potentially at risk from HAP, we compiled a list of counties with high co-incident primary solid-fuel use and percent of households below the FPL. We defined counties as high priority where ≥ 10% of the households use solid fuels as their primary heating source and where ≥ 20% of the households have incomes below the FPL (see Supplemental Material, Table S1 and Figure S1).

To develop an estimate of the risk for HAP from fugitive emissions released from household stoves in the United States, we searched the scientific literature from 1990 through 2013 for studies of U.S. households using solid fuels with the following search terms: household air pollution, indoor air pollution, air pollution, poverty, wood stove, and coal stove. The search revealed only a few studies that documented indoor air quality in U.S.-based homes using solid fuels as a primary heating source (Bunnell et al. 2010; Noonan et al. 2012a; Paulin et al. 2013; Robin et al. 1996; Ward et al. 2008, 2011). For each study, we attempted to determine the percentage of homes whose 24-hr average for PM_2.5_ (particulate matter with diameter ≤ 2.5 μm) exceeded the WHO recommended level of 25 μg/m^3^ (WHO 2005). We chose the WHO guideline for PM_2.5_ because the WHO standard is used for both indoor and outdoor air pollutant levels, as opposed to the daily National Ambient Air Quality Standard of 35 μg/m^3^ used for ambient air pollution alone (U.S. Environmental Protection Agency 2014). Of these, only three studies provided sufficient data to develop an estimate of the percentage of homes with PM_2.5_ levels that exceed the WHO 24-hr standard (Bunnell et al. 2010; Noonan et al. 2012a; Ward et al. 2011). The study by Bunnell et al. (2010) was conducted near Shiprock, New Mexico, on the Navajo Nation, and 19 homes were surveyed. The study by Noonan et al. (2012a) was conducted in Libby, Montana, and 26 homes were surveyed. The study by Ward et al. (2011) was conducted on a Nez Perce reservation in Idaho with 16 homes. The studies were conducted in geographic areas or populations with > 20% of the households below the FPL, one of the criteria for the “high priority” counties listed in the Supplemental Material (U.S. Census Bureau 2012).

However, two factors may potentially confound the interpretation of the high indoor PM_2.5_ levels: indoor tobacco smoke and ambient air pollution. The studies by Ward et al. (2011) and Noonan et al. (2012a) excluded homes with self-reported smokers; and Bunnell et al. (2010) tracked whether cigarette smoking occurred during a study period, and no smoking was reported during the winter heating season. These steps minimize or eliminate the possible confounding influence of environmental tobacco smoke (ETS). Regarding the indoor contributions from ambient air pollution, the two studies that changed out stoves and repeated PM_2.5_ levels found that the reductions in indoor PM_2.5_ were not influenced by changes in ambient levels (Noonan et al. 2012a; Ward et al. 2011). Additionally, the studies that measured ambient PM_2.5_ levels found that indoor concentrations were much higher than outdoor concentrations during sampling periods, suggesting that contributions from ambient pollution to indoor air pollution were likely small. Although excluding homes with ETS allows for a more specific estimate of the health impacts of burning solid fuels, it may well underestimate the number of households with indoor air quality above the WHO standards in our analysis, because burning of solid fuels and tobacco use likely overlap in many households (Paulin et al. 2013). Thus, the three studies selected to estimate the number of households at potential risk from fugitive emissions are the best currently available; they address the impact of tobacco smoke and ambient pollution in a consistent and logical manner.

## Results

**Figure 1:**
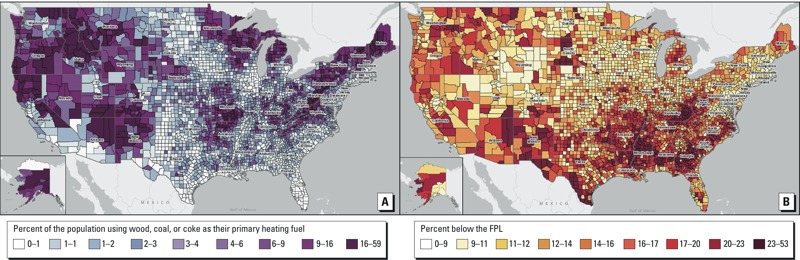
(*A*) Map of solid-fuel use by county in the United States, shown as the percentage of occupied housing units using wood, coal, or coke as the primary heating fuel (range, 0–59 in nine quantiles). (*B*) Map showing the percentage of people below the federal poverty level (FPL) in nine equal quantiles. Data from ACS 5 Year Estimate 2006–2010 (U.S. Census Bureau 2012).

The mapping exercise in our study reveals that solid-fuel use appears concentrated in the northern and western regions of the United States with some pockets of high use in the Midwest and Appalachian regions (Figure 1). In the United States, household solid fuel is used predominantly for heating, and the mapping of the U.S. Census data reflects areas of more temperate climate with seasonal needs for heating. Many areas of poverty consistently overlap with areas of solid-fuel use (Figure 1A), although high poverty tends to be concentrated in the South, where use of solid fuels for heating is much lower (Figure 1B). Most notably, areas of solid fuels use and poverty coexist in Southwest and Central Alaska; the Four Corners region of New Mexico, Arizona, Utah, and Colorado; Appalachia, particularly parts of Kentucky and West Virginia; and pockets throughout the Pacific Northwest. We identified 117 counties meeting the criteria for high co-incident primary solid-fuel use and percent of households below the FPL (see Supplemental Material, Table S1, Figure S1). Of these 117 counties, 107 are considered rural by the USDA rural–urban continuum score (USDA 2012).

As many as 600,000 people could be at risk for ill health due to high-level HAP exposure in the United States (Table 1). We calculated the number of people exposed to risk from HAP by multiplying the number of people who live in poverty and use solid fuel as their primary heating source (*n* = 900,000) by the range of values for the percentage of homes experiencing 24-hr average PM_2.5_ concentrations exceeding the WHO (2005) recommended level of 25 μg/m^3^ (between 53–65%, as shown in Table 1). Air pollution > 25 μg/m^3^ has been shown to negatively impact human health (WHO 2005). In this calculation, we assumed that the measurements of HAP were taken within homes representative of homes below the FPL. Unfortunately, household income was not specifically reported in any of the three studies used to establish the percent of homes exceeding the WHO indoor standard (Bunnell et al. 2010; Noonan et al. 2012a; Ward et al. 2011). However, U.S. Census data reveal that each of the studies was conducted in a geographic areas or populations where ≥ 20% of the population lives below the FPL (Navajo Reservation, 38%; Libby, Montana, 22%; Nez Perce Reservation, 15% for all residents and 22% among Native Americans, the study population) (Bunnell et al. 2010; Noonan et al. 2012a; U.S. Census Bureau 2012; Ward et al. 2011).

**Table 1:** Estimate of the number of people at risk for ill health from HAP due to burning solid fuels in the United States.

No. of people	Source
Approximately 6.5 million people in the United States live in homes heated primarily by wood, coal, or coke.	U.S. Census ACS 2006–2010, PUMS (U.S. Census Bureau 2012)
Of these, about 900,000 live below the FPL.	U.S. Census ACS 2006–2010, PUMS (U.S. Census Bureau 2012)
Indoor air quality studies have shown that 53–65% of homes in poorer areas that heat primarily with wood, coal, or coke exceed the WHO 24-hr particulate matter guidelines (2005).	53%: Ward et al. 2011, Nez Perce Reservation, Idaho (no. of homes = 16); 58%: Bunnell et al. 2010, Navajo Nation (no. of homes = 19); 65%: Noonan et al. 2012a, Libby, Montana (no. of homes = 26)
Generalizing the range of 53–65% to 900,000 is roughly 500,000–600,000 people living in homes that exceed the WHO 24-hr particulate matter guidelines (2005).	Air Quality Guidelines for particulate matter, ozone, nitrogen dioxide, and sulfur dioxide (WHO 2005)
Conclusion: 500,000–600,000 low-income people in the United States are exposed to HAP from burning solid fuels for residential heating.

## Discussion

The burning of solid fuels for heating occurs seasonally in large regions of the United States, and HAP can be found in rural areas with a history of poverty (Barry et al. 2010; Bulkow et al. 2012; Bunnell et al. 2010; Noonan et al. 2012b; Ward et al. 2011). In this study, we present a geographic distribution of household solid-fuel use and an estimate of the number of low-income Americans at risk from exposure to HAP. Given the available evidence, we estimated that 500,000–600,000 low-income Americans are at risk for adverse health effects as a result of HAP. To our knowledge, this is the first study to estimate of the number of low-income Americans potentially exposed to HAP from household solid-fuel use, using the U.S. Census and extrapolating the number of households at risk from HAP based on the existing scientific literature. Although these studies employed nonrandom sampling, were small in size, and did not record demographic information such as household size or income, they are the best available evidence (Bunnell et al. 2010; Noonan et al. 2012a; Ward et al. 2008, 2011). Without specific household income information, it is not possible to verify whether all of the homes included in these studies were indeed representative of households with incomes below the FPL. The nonrandom selection of households for monitoring may tend to overestimate the likelihood of fugitive emissions to the extent that study participation may be biased to households with higher emissions. However, our estimate of Americans at risk from household solid-fuel use is likely conservative. We did not estimate the total exposure from solid-fuel use by including the contribution of vented emissions to ambient air pollution, as was done for household air pollution in the global comparative risk assessment report (Lim et al. 2012). Furthermore, we did not attempt to include the approximately 9 million households that use solid fuels as a secondary source of heating, because peer-reviewed data currently do not exist on the level of fugitive emissions from stoves and fireplaces among secondary users (U.S. Energy Information Agency 2012). The selection of the WHO 24-hr average recommendation for PM_2.5_ may also have resulted in an underestimate of those at risk because the WHO has recommended an annual average of 10 μg/m^3^ for PM_2.5_; and if measurements in the sampled homes indicate standard practices, then more homes might have exceeded this lower standard. However, each of the studies upon which this analysis relied reported its findings as 24-hr average PM_2.5_. Ideally, future estimates of adverse health risks should be based on direct measurements as part of a coordinated set of regional stove changeout programs with enough households to achieve sufficient statistical power to more accurately extrapolate the burden of disease risk nationally.

The priority counties that we identified were predominantly rural, 107 of 117, according to the USDA county typology codes (USDA 2012). These counties, on average, also have higher infant mortality, and their average death rate from chronic lower respiratory disease was nearly twice the national average (Health Indicators Warehouse 2013). Although the association with poor health outcomes is purely ecological—it cannot be verified whether homes with high solid-fuel use also have a higher burden of disease—studies linking HAP and markers of respiratory health have been conducted in several of the counties we identified (Barry et al. 2010; Bulkow et al. 2012; Morris et al. 1990; Noonan et al. 2012b; Robin et al. 1996). This strengthens our confidence that despite the risk for ecologic fallacy inherent in such comparisons, there may be an association between HAP and ill health in these counties. Future research on HAP and changeout programs should focus on these priority counties or counties with similar characteristics in order to be most effective.

There are differences with HAP in the United States compared with HAP in LMIC (Naeher et al. 2007). First, heating in the United States is usually a seasonal need, so one might expect overall exposures from solid-fuel use to be lower than those in LMIC where the predominant daily energy need is for cooking. Second, solid-fuel use in the United States also occurs among non–low-income families as an optional and supplemental source of fuel for heating or simply for recreational use in the home; additionally some non–low-income families may choose solid-fuel use as their primary heating fuel for additional real or perceived benefits (e.g., carbon neutrality, cost stability) (Allen et al. 2009; Naeher et al. 2007). However, even in non–low-income homes with seasonal wood fuel use, the levels may not be entirely safe, although studies remain to be done to provide accurate estimates. There is ample evidence that woodsmoke contributes significantly to ill health in airsheds surrounding such cities as Seattle, Washington (Naeher et al. 2007). This demonstrates the large “neighborhood” effect of inefficient woodstoves and suggests that the unsafe use of indoor solid fuels for heating is not limited to households in poverty. Despite these differences, there are similarities between solid-fuel use in the United States and that in LMIC. For example, as in LMIC, solid fuels may be the only fuel source available in populations with a lower socioeconomic status (SES). For those living in a rural U.S. community with a lower SES, the release of fugitive emissions into the households may occur more often because of the increased financial burden associated with purchase of more efficient heating stoves or the routine maintenance/inspection necessary to assure adequate ventilation of emissions. As is the case globally, the individuals and families who bear the greatest risk are also the ones who can least afford to make health-promoting changes to counteract these risks. HAP is an environmental justice issue both domestically and globally. Various types of solid fuels may be used in households in the United States and globally, some of which have different impacts on health outcomes. For example, household use of coal, which remains common in China, is associated with a higher risk of lung cancer than wood-fuel use alone (Straif et al. 2006). Although most people in the United States who use solid fuels as their primary heating source use wood (6.1 of 6.5 million), the remainder who use coal and coke (U.S. Census Bureau 2012) may be more likely to have health effects similar to those elsewhere in the world who use coal as their primary household energy source.

Health risks from household solid-fuel use are not limited to household exposures. In rural areas, communities with a high proportion of household solid-fuel use with emissions vented outside may significantly contribute to ambient air pollution and place entire communities and regions at risk for adverse health outcomes (Johnston et al. 2013; Larson et al. 2004; Noonan et al. 2012b; Sheppard et al. 1999; Ward and Lange 2010). In fact, the Libby, Montana, woodstove changeout program reduced average winter ambient PM_2.5_ by 28%; and in the small subset of homes in which household monitoring occurred, 24-hr average household PM_2.5_ was also reduced by 53% (Noonan et al. 2012a, 2012b). These improvements in air quality were associated with reductions in respiratory symptoms in children, including parent-reported respiratory infections (Noonan et al. 2012b). Thus, adverse health effects result not only from the direct exposure to HAP, but also from its contribution to ambient pollution in the wider community. These findings are also consistent with the recent report by Lim et al. (2012), which noted that almost 0.5 million additional deaths from HAP occur each year globally as a result of the impact of HAP on outdoor air pollution.

Especially when HAP is viewed in the context of poverty, confounding factors can also adversely affect health, including higher risk for infections, poor nutrition, and other environmental health risks. Smoking and ETS within the home also represent a significant challenge in studying the health effects of HAP (Noonan and Ward 2007; Paulin et al. 2013). However, the effect of HAP can often still be found even in the context of confounders. For example, in Alaska Native villages where smoking rates among women are as high as 60% (Kim et al. 2010), having a wood or coal stove in the house was a significant independent risk factor for acute lower respiratory tract infection in children < 3 years of age (Bulkow et al. 2012). As with all research on environmental exposures, it is difficult to separate the effects of multiple exposures. Future studies should seek to determine the effects of HAP through careful exposure monitoring. Examples of such studies include the recently announced randomized controlled trial in the United States using asthma as an outcome, which is beginning to publish results, and a randomized trial using air filtration and measuring endothelial function in a community affected by woodsmoke (Allen et al. 2011; McNamara et al. 2013; Noonan and Ward 2012).

The 500,000–600,000 low-income individuals potentially at risk from HAP in the United States merit the attention of the scientific and policy-making communities. The number of people affected by HAP in the United States may be modest compared with those affected by other sources such as traffic-related air pollution (Health Effects Institute 2010). This does not negate the fact that those affected by HAP tend to have high exposures for months at a time and, as occurs with HAP elsewhere in the world, often have limited options to reduce the exposures. To enhance the accuracy of this estimate, additional studies are needed including detailed indoor and outdoor exposure monitoring of households and communities that commonly use solid fuels for heating, so that reductions in exposure can be verified and correlated with health outcomes. Such work will provide the necessary data to elucidate the health effects across the life cycle and better account for differences in disease severity and societal cost based on the specific subpopulations most affected. Future studies may provide impetus for public policy changes to foster improved air quality and, we hope, improvements in health of underserved and vulnerable populations in the rural United States. Potential health risks exist from household burning of solid fuels in wealthier countries, particularly in poor areas where households may lack access to clean-burning technologies. Investments by developed countries such as the United States to help solve these environmental risks in LMIC, where 40% of the world’s population lives with far greater household exposures, may also prove valuable to address solid-fuel use in high-poverty areas at home.

## Supplemental Material

(426 KB) PDF
