# Supplementary material for: Estimating the Number of Low-Income Americans Exposed to Household Air Pollution from Burning Solid Fuels
Source: Environ Health Perspect. 2014 May 9;122(8):806–10. doi: 10.1289/ehp.1306709 (PMC4123020; doi:10.1289/ehp.1306709)
Supplement: (426 KB) PDF [file ehp.1306709.s001.pdf]

Environmental Health Perspectives doi:10.1289/ehp.1306709

Supplemental Material

Estimating the Number of Low-Income Americans Exposed to Household Air Pollution from Burning Solid Fuels

Derek K. Rogalsky, Pauline Mendola, Tricia A. Metts, and William J. Martin II

Table of Contents **Page**

Table S1. High priority counties for research on health effects of HAP in the US **2**

Figure S1. Map of high priority counties **7**

References **8**

Table S1. High priority counties for research on health effects of HAP in the US.

County, state	Percent of households using wood, coal, or coke as primary heating fuel[a]	Percent below the Federal Poverty Level[b]	Deaths from chronic lower respiratory disease per 100,000[c]	Infant mortality per 1,000 live births[d]	Rural-urban continuum score[e]
Lake and Peninsula Borough, Alaska	12	21	NA	NA	9
Wade Hampton Census Area, Alaska	17	31	54	12	9
Yukon-Koyukuk Census Area, Alaska	47	24	NA	8	8
Apache County, Arizona	58	34	22	8	6
Navajo County, Arizona	33	24	38	8	4
Fulton County, Arkansas	21	20	114	8	9
Montgomery County, Arkansas	16	20	103	4	8
Newton County, Arkansas	32	23	60	5	9
Pike County, Arkansas	11	21	112	13	9
Polk County, Arkansas	16	20	124	9	7
Randolph County, Arkansas	15	20	82	8	7
Scott County, Arkansas	16	24	57	6	6
Searcy County, Arkansas	29	23	80	8	9
Sharp County, Arkansas	14	22	83	7	7
Stone County, Arkansas	23	23	83	5	9
Van Buren County, Arkansas	17	23	99	6	8
Del Norte County, California	19	22	64	8	7
Tehama County, California	20	20	84	5	4
Costilla County, Colorado	16	28	NA	17	9
Huerfano County, Colorado	13	21	112	6	6
Lake County, Colorado	11	22	NA	12	7
Saguache County, Colorado	24	24	NA	6	9
Latah County, Idaho	12	22	28	5	4
Lemhi County, Idaho	39	20	71	5	7
Owyhee County, Idaho	11	22	44	10	2

County, state	Percent of households using wood, coal, or coke as primary heating fuel[a]	Percent below the Federal Poverty Level[b]	Deaths from chronic lower respiratory disease per 100,000[c]	Infant mortality per 1,000 live births[d]	Rural-urban continuum score[e]
Orange County, Indiana	11	20	70	10	6
Breathitt County, Kentucky	13	33	108	9	7
Carter County, Kentucky	12	21	74	8	6
Casey County, Kentucky	20	28	73	7	9
Cumberland County, Kentucky	15	27	87	6	9
Elliott County, Kentucky	18	37	80	5	9
Fleming County, Kentucky	14	20	78	4	7
Hart County, Kentucky	10	24	77	5	8
Jackson County, Kentucky	10	33	71	5	9
Lee County, Kentucky	10	32	120	7	9
Leslie County, Kentucky	11	25	98	9	9
Lewis County, Kentucky	20	28	89	6	8
Lincoln County, Kentucky	12	22	97	5	7
McCreary County, Kentucky	14	35	96	10	9
Menifee County, Kentucky	14	20	78	6	9
Monroe County, Kentucky	14	27	56	5	9
Morgan County, Kentucky	10	24	66	6	7
Robertson County, Kentucky	14	25	NA	NA	8
Wayne County, Kentucky	12	27	88	7	7
Wolfe County, Kentucky	16	42	120	7	9
Washington County, Maine	14	20	87	5	7
Clare County, Michigan	11	22	92	9	7
Houghton County, Michigan	11	22	51	5	5
Keweenaw County, Michigan	20	20	NA	NA	9
Lake County, Michigan	23	20	103	12	8
Mecosta County, Michigan	10	21	52	9	6
Oscoda County, Michigan	24	20	108	6	9

County, state	Percent of households using wood, coal, or coke as primary heating fuel[a]	Percent below the Federal Poverty Level[b]	Deaths from chronic lower respiratory disease per 100,000[c]	Infant mortality per 1,000 live births[d]	Rural-urban continuum score[e]
Beltrami County, Minnesota	10	20	45	6	7
Mahnomen County, Minnesota	13	24	NA	7	8
Choctaw County, Mississippi	10	21	74	9	9
Bollinger County, Missouri	19	20	69	12	9
Carter County, Missouri	23	20	117	8	9
Dade County, Missouri	14	21	76	5	8
Douglas County, Missouri	28	22	89	6	6
Howell County, Missouri	16	20	84	6	7
Iron County, Missouri	14	22	105	8	6
Knox County, Missouri	11	21	97	6	9
Oregon County, Missouri	23	24	50	6	9
Polk County, Missouri	11	22	89	6	2
Reynolds County, Missouri	24	21	87	15	9
Ripley County, Missouri	13	24	102	8	9
Shannon County, Missouri	30	23	76	13	9
Texas County, Missouri	24	22	91	11	9
Washington County, Missouri	16	21	53	7	1
Wayne County, Missouri	15	20	135	14	9
Wright County, Missouri	24	25	77	8	6
Deer Lodge County, Montana	11	21	119	8	7
Glacier County, Montana	13	25	47	9	7
Lake County, Montana	18	22	59	9	6
Sanders County, Montana	41	21	74	8	8
Keya Paha County, Nebraska	19	23	NA	NA	9
Cibola County, New Mexico	22	24	48	6	6
Guadalupe County, New Mexico	15	28	NA	9	7
Hidalgo County, New Mexico	12	23	NA	7	7

County, state	Percent of households using wood, coal, or coke as primary heating fuel[a]	Percent below the Federal Poverty Level[b]	Deaths from chronic lower respiratory disease per 100,000[c]	Infant mortality per 1,000 live births[d]	Rural-urban continuum score[e]
McKinley County, New Mexico	36	33	20	10	4
Rio Arriba County, New Mexico	18	20	34	4	6
San Juan County, New Mexico	15	21	40	6	3
San Miguel County, New Mexico	28	25	47	7	6
Socorro County, New Mexico	18	27	65	3	6
Alleghany County, North Carolina	11	26	88	8	9
Clay County, North Carolina	11	21	79	9	9
Swain County, North Carolina	13	22	78	5	8
Adams County, Ohio	10	23	80	7	6
Vinton County, Ohio	10	20	76	9	9
Adair County, Oklahoma	15	27	78	9	6
Delaware County, Oklahoma	12	21	75	6	6
Pushmataha County, Oklahoma	14	27	85	8	9
Baker County, Oregon	18	20	78	7	7
Jefferson County, Oregon	20	20	56	9	6
Sherman County, Oregon	16	20	NA	NA	9
Jackson County, South Dakota	11	30	NA	16	8
Mellette County, South Dakota	12	27	NA	19	9
Todd County, South Dakota	11	49	NA	12	9
Bledsoe County, Tennessee	10	23	73	5	8
Fentress County, Tennessee	13	25	96	11	9
Hancock County, Tennessee	27	30	94	9	8
Johnson County, Tennessee	11	24	89	13	6
Meigs County, Tennessee	10	25	81	9	8
Perry County, Tennessee	11	24	64	6	8
Van Buren County, Tennessee	10	25	NA	9	9
San Juan County, Utah	34	26	NA	4	7

County, state	Percent of households using wood, coal, or coke as primary heating fuel[a]	Percent below the Federal Poverty Level[b]	Deaths from chronic lower respiratory disease per 100,000[c]	Infant mortality per 1,000 live births[d]	Rural-urban continuum score[e]
Buchanan County, Virginia	11	22	100	11	9
Ferry County, Washington	52	21	85	11	9
Klickitat County, Washington	22	20	62	4	6
Okanogan County, Washington	24	20	64	6	6
Braxton County, West Virginia	18	21	88	7	8
Clay County, West Virginia	17	24	96	6	2
McDowell County, West Virginia	17	33	127	12	7
Summers County, West Virginia	22	22	87	13	7
Webster County, West Virginia	33	23	93	7	9
Menominee County, Wisconsin	21	32	NA	8	8
Sawyer County, Wisconsin	16	20	73	7	9

117 high priority counties out of 3144.

[a]Percent of Households Using Wood, Coal, or Coke as Primary Heating Fuel: Data from American Community Survey 2006-2010. [b]Percent of Households Below Federal Poverty Level: Data from American Community Survey 2006-2010. The Federal Poverty Level (FPL) is defined by using set income levels adjusted for inflation and family size; for example, the FPL in 2011 for a family of four with two children was 22,811. [c]Deaths from Chronic Lower Respiratory Disease: Defined as deaths due to ICD-10 codes: J40-J47 per 100,000 occurring from 2006-2010, which includes Chronic Obstructive Pulmonary Disease and Asthma. National Vital Statistics System-Mortality, collected by Centers for Disease Control and Prevention, National Center for Health Statistics. Accessed through http://HealthIndicators.gov. [d]Infant Mortality: Infant Mortality expressed in deaths within the first year of life for every 1,000 live births. Community Health Status Indicators 1996-2005. Accessed through http://wwwn.cdc.gov/CommunityHealth/homepage.aspx?j=1. [e]Rural-Urban Continuum Score: Range 1=Metro Area to 9=Completely Rural. Source: 2004 County Typology Codes (US Department of Agriculture 2012).

NA-Not enough data to calculate a rate.

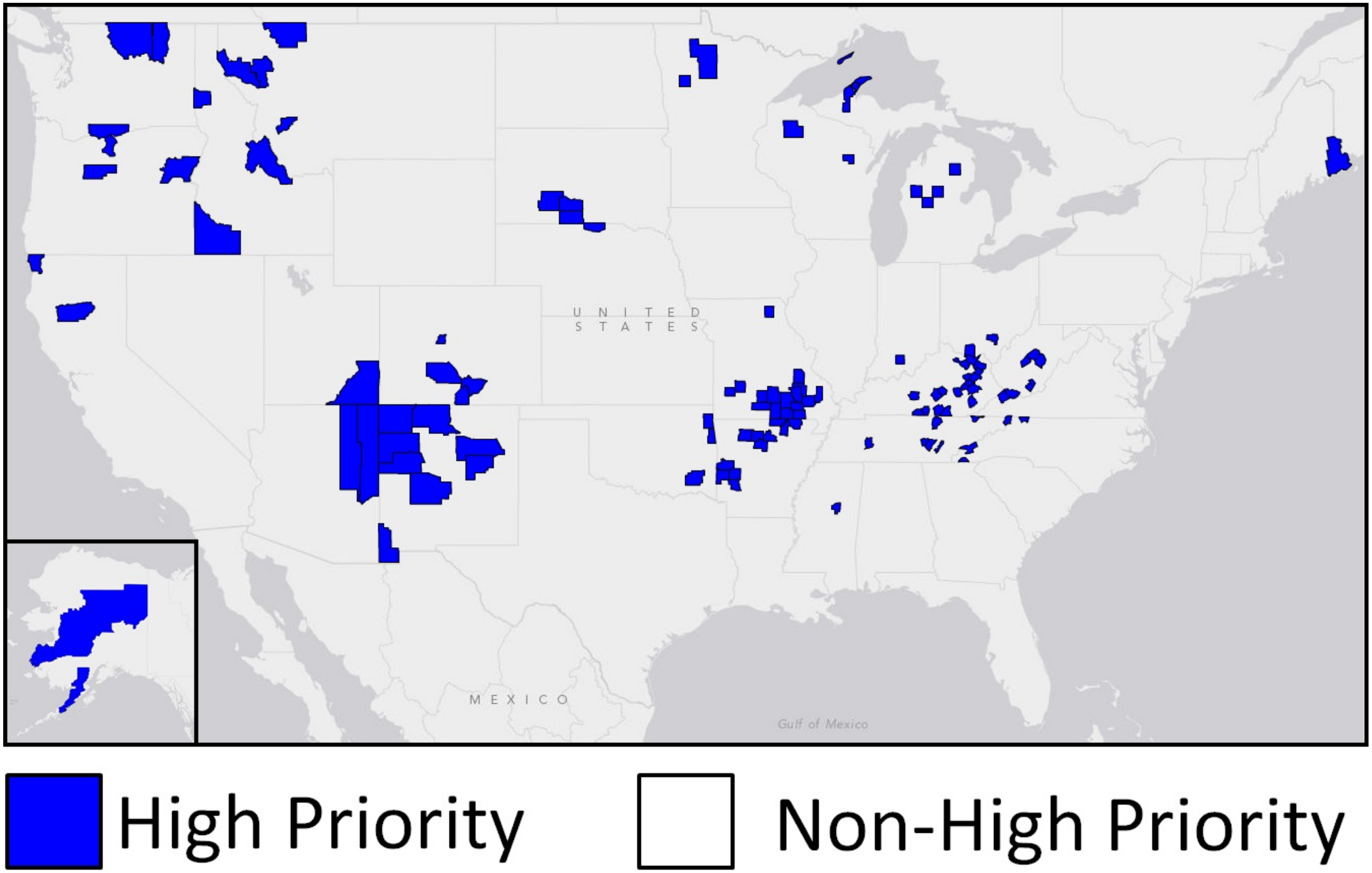

Figure S1. Map of high priority counties. High priority is defined as >20% of population below the Federal Poverty Level or greater and 10% of greater of the households using wood, coal, or coke as their primary heating fuel.

References

US Census Bureau. 2012. http://dataferrett.census.gov/ https://www.census.gov/acs/www/data_documentation/public_use_microdata_sample/ https://www.census.gov/acs/www/ [accessed 12 January 2014].

Health Indicators Warehouse. 2013. http://healthindicators.gov/ National Vital Statistics System-Mortality, collected by Centers for Disease Control and Prevention and National Center for Health Statistics. [Accessed 12 January 2014].

US Department of Agriculture. 2012. http://ers.usda.gov/data-products.aspx [Accessed 12 January 2014].
